# Resistant Catatonia in a 10-year-old Child: A Case Report

**DOI:** 10.31729/jnma.8152

**Published:** 2023-05-31

**Authors:** Amit Jha

**Affiliations:** 1 Child and Adolescent Mental Health Unit, Kanti Children's Hospital, Maharajgunj, Kathmandu, Nepal

**Keywords:** *benzodiazepines*, *case reports*, *catatonia*, *electroconvulsive therapy*

## Abstract

Catatonia is a psycho-motor disorder associated with various psychiatric, neurological, and medical illnesses. It is due to alteration in GABAergic circuits and basal ganglia. Management includes identifying the underlying cause and handling complications with supportive treatment. It can cause life-threatening complications like dehydration and cardiac arrest. The risks are more in children and adolescent populations. Benzodiazepines and electro-convulsive therapy are treatment modalities. In this case report we discuss about a child who was resistant to both lorazepam and electroconvulsive therapy. Resistance to both first-line management is a rare phenomenon. We were able to manage with a combination of antipsychotics and antidepressants. Catatonia in children may respond late to treatment. Symptomatic treatment, ruling out organic causes, and judicious use of pharmacotherapy can be beneficial in resistant cases.

## INTRODUCTION

Catatonia is characterized by signs of psychomotor disturbances. Pediatric catatonia has been reported with schizophrenia, post-traumatic stress disorder, autism spectrum disorder and tic disorder. Mutism, negativism, posturing and waxy flexibility remains the most common symptoms. In pediatric population, withdrawn to self and rigidity are major presenting sign.

The prevalence of catatonia ranges from 0.6%-17%.^1^ Children with catatonia differs from adult in having more of psychomotor regression with history of neuro- developmental disorders and more co-morbidity.^1^ Electroconvulsive therapy, benzodiazepines and treatment of underlying cause remains the gold standard treatment.^2,3^ Clinicians have used anticonvulsant, memantine and zolpidem in resistant cases.

## CASE REPORT

A 10-year-old female child studying in class 4 with milestones comparable to other children her age, presented with acute onset, rapidly progressing illness of 2.5 months beginning with fever and subsequently characterized by decreased sleep, fearfulness, clinging to parents, suspiciousness, and vivid visual hallucinations. Her interaction decreased and started remaining withdrawn. She had involuntary movements of limbs lasting for 30-40 min with a total of 5-6 episodes over 2 months period.

Within a month of illness, she was further withdrawn, would not speak, and depended on family members for activities of daily living. No family history of psychiatric illness, or other neuro-developmental disorders. After a routine initial work-up, special investigations were done to identify a possible cause. Investigations included serum and CSF encephalitis panels as well as anti-bodies studies. Electroencephalogram showed no seizures. MRI brain reported a normal scan whereas PET-MRI was suggestive of non-specific catatonic features since none of the investigations and imaging studies revealed any abnormality she was treated symptomatically.

She received two trials of lorazepam up to 6 mg, over a month period without improvement. She had received sodium valproate, syndopa, selegeline, clobazam, baclofen, and trihexyphenidyl at different dose ranges without improvement. She also underwent plasmapheresis including five dosages of pulse methylprednisolone. After a month of stay in neurology with minimal improvement, she was referred to the Department of Child and Adolescent Psychiatry.

She was cachexic, weighing 25 kg with in-situ Ryle's tube and Foley catheter. She had lost 12 kg in 3 months. Clinically, she had immobility, rigidity, and withdrawn behavior. On Kirby's, her posture was passive with active negativism on re-positioning. She had a fixed gaze with active resistance. She would not respond to any command with no emotional responsiveness and mute speech. Pediatric Catatonia Rating Scale (PCRS) at admission was 19 with items positive for stupor, staring, negativism, rigidity, withdrawal, mutism, and refusal to eat and drink.

She was planned for electro-convulsive therapy (ECT) after consent of family members. She was admitted to highly intensive care unit (HICU) of ward with 24-hour nursing support, intravenous fluids, and feeding by Ryle's tube with input and output charting and vitals monitoring. Two hourly repositioning of posture was done with an air-filled mattress, her joints were supported by water-filled bags to prevent stress ulcers. ECT was started at the frequency of 3 per week with a total of seven ECTs. In between trials of injection, lorazepam was given, with no improvement. After four sessions of ECT, PCRS score decreased to 14.

She was started on both antipsychotics and antidepressants. Tab. olanzapine was started at 2.5 mg which was increased to 10 mg and tab. sertraline was started at 12.5 mg which was increased to 50 mg over a week period. With no further improvement by ECT after the seventh session, it was decided to stop. She was discharged after a month. At the time of discharge, her PCRS score was 12, with improvement in spontaneous eye opening, and staring, a decrease in active negativism and a decrease in rigidity. She was discharged with liaison at local medical college for the continuation of subsequent treatment. At the time of discharge, she was on Ryle's tube feeding with Foley catheterization and on 50 mg of sertraline and 10 mg of olanzapine in divided doses.

Follow-up was done after a month, with improvement noted in rigidity, withdrawal behavior, staring, mutism, and negativism. Her Ryle's tube and Foley catheter were out, changing of gaze was present, and was receiving the oral feed. She was maintained on the same dosage of medication as of discharge and had gained 2 kg body weight, however, she was still bedridden. On second month of discharge, she continued to improve. There was spontaneity in speech and mobility with adequate sleep and feeding habit. At third month of discharge, she was independent in activities of daily living. She was able to brush, bathe, eat, defecate, and urinate independently. She was continued on 10 mg of olanzapine and 50 mg of sertraline without any side effects.

Follow-up was done after 6 months during which the child was interviewed in-depth. The interview revealed stressors prior to the onset of the illness. She also had obsessive-compulsive symptoms; however, they were not on a syndromal level. It was conceptualized to be effective in origin and was continued with the same combination treatment. The child continues to maintain well after a year of discharge.

## DISCUSSION

Catatonia causes disturbance in psychomotor functioning with hypoactivation of gamma-aminobutyric-acid functioning. As benzodiazepines like lorazepam's primary target is the same receptor, it is indicated.^4^ Another option includes ECT, which is safe with reversible cognitive side effects.^5^ Treating the underlying cause of catatonia is the next management approach. Catatonia effectively responds to lorazepam and ECT, and lorazepam has a remission rate of 79% for symptoms of catatonia within 48-72 hours.^6,7^

The following case presentation is unique because no organic or clear-cut psychiatric symptoms were identified. The cause of catatonia was unknown after intensive investigations and treatment. DSM-5 and ICD-11 define catatonia as a separate entity and a syndrome in itself. It can be secondary to a general medical condition, due to organic conditions, or due to psychiatric illnesses. It can be due to affective or psychotic etiologies, drugs, autoimmune conditions, seronegative encephalitis, and neurodevelopmental conditions. Pediatric catatonia is commonly associated with trauma, neurodevelopmental disorders, anti-NMDA receptor encephalitis, and other conditions.^4^

In our case, we excluded possible organic causes with investigative and imaging studies. During the later part of treatment, child was treated in line of seronegative autoimmune encephalitis with no apparent improvement, as the dilemma continued to persist. Such instances are common in pediatric patients. In some cases, authors have tried high dosages of zolpidem, which is a benzodiazepine with a positive allosteric modulating effect on GABA-A receptors with significant improvement.^8^

If lorazepam, ECT, antipsychotics, and antidepressants don't work, where etiology is uncertain, authors have suggested using anticonvulsants like valproate, and carbamazepine. Memantine, a non-competitive antagonist at NMDA receptor has also been mentioned in the literature.^9^ In our case one of the reasons why the combination of anti-depressant and anti-psychotic worked maybe postulated to a child's higher threat perception during the beginning of illness which could have been a pointer to wards affective illness. It should also be noted that children and adolescent population diagnosed as having seronegative autoimmune encephalitis with features of catatonia should be treated for catatonia and are better served with a diagnosis of catatonia under evaluation even if no psychiatric cause to it has been found.^10^ Rather than immunotherapies, judicious use of antipsychotics and antidepressants can work better if adequate dosage and duration of these medications are given even if first-line treatment like lorazepam and ECT fails.

Pediatric catatonia once considered as a rare entity is a common psychiatric emergency owing to recent publications and renewed interest among academicians. It can occur with a large number of identifiable and unidentifiable medical and psychiatric conditions. Commonly underdiagnosis results in a delay in treatment and reversion of complications associated with it which can be life-threatening. Management can be difficult with many cases not responding to the standard treatment protocol, in which case, taking recommendations from adult literature and following some case reports and case series can be a way to approach.
